# To Compare the Safety and Efficacy of Conscious Sedation with Dexmedetomidine and Propofol Infusion for Endoscopic Discectomy

**DOI:** 10.5152/TJAR.2022.21381

**Published:** 2022-12-01

**Authors:** Kalyani Nilesh Patil, Shalini P. Sardesai

**Affiliations:** 1 Department of Anaesthesiology and Critical Care, Smt. Kashibai Navale Medical College and General Hospital, Pune, India

**Keywords:** Conscious sedation, dexmedetomidine, outpatient anaesthesia, postoperative analgesia, propofol

## Abstract

**Objective:** Endoscopic discectomy is a minimally invasive, day care spine surgery. Patient comfort is of utmost importance as it is performed under local anaesthesia and in prone position. Propofol and dexmedetomidine are titrable and short-acting and commonly used for conscious sedation. The objectives of the study are to study the effect of dexmedetomidine and propofol infusion on cardiorespiratory parameters and to evaluate the efficacy of dexmedetomidine and propofol infusion for conscious sedation.

**Methods:** This is a prospective, randomized, patient-blinded study. Sixty adult patients were randomly recruited to 2 groups of 30 each, to receive an infusion of dexmedetomidine or propofol, titrated to bispectral index score 70-80. The intra-operative cardiorespiratory parameters and level of sedation, postoperative visual analogue scale score, time to discharge from post-anaesthesia care unit, and patient satisfaction were monitored.

**Results:** Both groups were comparable with respect to demographic parameters and surgical duration. The heart rate was significantly lower with dexmedetomidine whereas the intraoperative mean arterial pressure was higher with dexmedetomidine. Though the level of intraoperative sedation was higher with propofol, the respiratory parameters were comparable. Postoperative visual analogue scale score was significantly higher with propofol.

**Conclusion:** Dexmedetomidine and propofol provide adequate sedation without any cardiorespiratory compromise when used for conscious sedation for minimally invasive spine surgeries performed in prone position. Dexmedetomidine provides an added advantage of postoperative analgesia and better patient satisfaction.

**Main Points**

- Endoscopic spine surgeries are increasingly being performed.
- Such surgeries performed in prone position demand patient comfort as well as patency of airway and preservation of spontaneous respiration.
- Titrable hypnotic agents that are devoid of respiratory depression are the choice of drugs.

## Introduction

α2-adrenergic receptor agonists have been successfully used in various clinical settings due to the diversity of their actions which include sedation, analgesia, anxiolysis, perioperative sympatholysis, cardiovascular stabilizing effects, reduced anaesthetic requirements, and preservation of respiratory function.^1^ Since 1999, dexmedetomidine has been approved by the Food and Drug Administration (FDA) for use in humans, for short-term sedation, and as analgesia (lasting for less than 24 hours) in the intensive care unit.

Propofol is an FDA-approved anaesthetic agent used for induction and maintenance of general anaesthesia and for sedation in ventilated patients.^2^ It is classified as an ultrashort-acting hypnotic agent. Propofol possesses sedative, amnestic, and hypnotic properties but provides minimal levels of analgesia.^3^

“Conscious sedation” is defined as a drug-induced depression of consciousness during which patients respond purposefully to verbal commands, either alone or accompanied by light tactile stimuli. The spontaneous respiration is adequate, and no interventions are required to maintain the airway patent.^4^ The degree of sedation should be titrated to achieve patient comfort as well as procedural success. Patients may require different levels of sedation for the same procedure and the same patient may attain varying levels of sedation during a single procedure.

We aim to study the effect of dexmedetomidine and propofol infusion on cardiorespiratory parameters and to evaluate their efficacy for conscious sedation.

## Methods

After obtaining clearance from institutional ethics committee, a prospective, randomized, patient-blinded study was carried out on 60 patients of either sex, in the age group 18-60 years and American Society of Anaesthesiologists physical status I and II, undergoing elective endoscopic discectomy.

Patients on adrenoreceptor agonist or antagonist therapy, those with known hypersensitivity to local anaesthetics, bleeding disorders, obstructive sleep apnoea, body weight more than 50% of ideal body weight, pre-existing peripheral neuropathy, and pregnant or lactating women were excluded from the study.

Considering standardized effect size of 0.75 and 80% power (β), the sample size for each group was calculated as 30. Patients were randomly recruited to 2 groups of 30 each to receive an infusion of either dexmedetomidine or propofol, titrated to maintain bispectral index score (BIS) of 70-80.

Group D: IV dexmedetomidine as an initial loading dose, at the rate of 0.5-1 µg kg^−1^ over 10 minutes followed by maintenance dose at 0.5-1 µg kg^−1^ h^−1^.

Group P: IV propofol as an initial loading dose of 65-75 μg kg^−1^ min^−1^ for 10 minutes followed by maintenance dose of 12.5-75 µg kg^−1^ min^−1^.

Thorough preoperative assessment was done on previous day of surgery. The nature and safety of the procedure were explained and a written, valid, informed consent was obtained after explaining visual analogue scale (VAS) score to the patients.

On arrival in operation theatre, standard monitors like pulse oximeter, non-invasive blood pressure, and electrocardiogram were attached and baseline values were noted. Intravenous access was secured with 20 G cannula for premedication and maintenance fluid administration. Oxygen supplementation was done with nasal prongs at 2 L min^−1^ throughout the procedure. End-tidal CO_2_ (EtCO_2_) was sampled through one port of the cannula.

The adequacy of conscious sedation was based on an electroencephalogram BIS of 70-80. The skin was cleaned with alcohol and left to dry. The BIS sensor was placed on the forehead and temple using a frontal-temporal assembly, pressed for 5 seconds, and skin-sensor connection was established.

Patients were premedicated with IV midazolam 0.03 mg kg^−1^ and IV fentanyl 2 µg kg^−1^ intravenously. All patients received IV glycopyrrolate 4 µg kg^−1^ and IV ondansetron 4 mg intravenously. Patients were then made prone. Axillary rolls were applied and all the pressure points were padded. The infusion of study drug was started through a separate 22 G intravenous cannula. Cardiorespiratory end points (heart rate, mean arterial pressure, arterial oxygen saturation, respiratory rate, and EtCO_2_) were noted at every 5-minute interval. On achieving the targeted BIS, surgery was commenced, and infusion doses were adjusted intraoperatively to maintain the BIS between 70 and 80.

Sedation was also assessed with Ramsay Sedation Score every 15 minutes till the end of the surgery. (1: anxious, agitated; 2: cooperative, oriented; 3: responsive to commands only; 4: brisk response to light glabellar tap or loud auditory stimuli; 5: sluggish response to light glabellar tap or auditory stimuli; 6: no response to light glabellar or auditory stimuli). The infusion was stopped after skin closure and the patients were made supine. The total duration of surgery was recorded.

The patients were monitored in the supine position till they obeyed simple verbal commands and were then shifted to post-anaesthesia care unit (PACU).

Postoperatively, the patients were monitored for cardio respiratory parameters, sedation, visual analogue scale (VAS) on a scale of 0-10, where 0 is no pain and 10 is worst imaginable pain, and post anaesthesia recovery score (0-10), every 15 minutes for 2 hours. Patient was discharged from PACU to the ward once 2 consecutive Aldrete score was ≥9. At 24 hours, patients were interviewed and patient satisfaction was noted. It was graded as 5-very satisfied, 4-satisfied, 3-neutral, 2-dissatisfied, 1-very dissatisfied.

### Statistical Analysis

The data were entered in MS Excel and was analyzed using Statistical Package for the Social Sciences software version 20 (IBM Corp.; Armonk, NY, USA) and Epi info version 7.2.4. Levene’s test for equality of variances was used and equal variances were assumed within the groups. Independent sample test (unpaired *t*-test) was used to test equality of means. Post hoc analysis was done using Tukey’s test by considering 5% margin of error (α). *P* ≤ .05 was considered significant.

## Results

There was no statistically significant difference in the demographic profile and the baseline values of haemodynamic variables between the 2 groups (Table 1).

**Figure 1.**
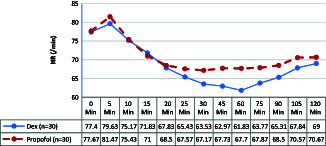
Comparison of intraoperative mean heart rate (HR).

**Figure 2.**
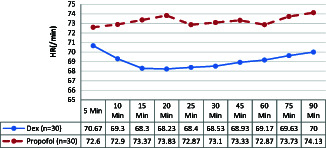
Comparison of postoperative mean HR.

The mean intraoperative and postoperative heart rate was significantly lower with dexmedetomidine as compared to propofol (*P* ≤ .002) (Figures 1 and  2).

**Figure 3.**
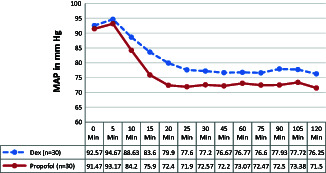
Comparison of intraoperative mean arterial pressure (MAP).

**Figure 4.**
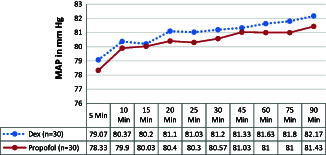
Comparison of postoperative mean MAP.

The intraoperative mean arterial pressure was significantly higher with dexmedetomidine (*P* ≤ .002) (Figure 3). There was, however, no significant difference postoperatively (Figure 4).

**Figure 5.**
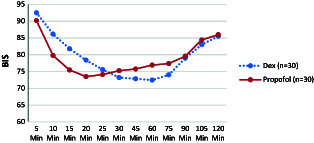
Comparison of intraoperative mean BIS. BIS, bispectral index score.

**Figure 6.**
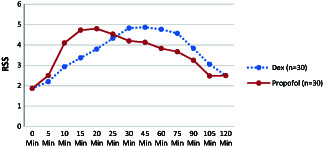
Comparison of intraoperative mean Ramsay Sedation Score.

The level of intraoperative sedation (Figures 5 and  6) was higher with propofol. The deeper level of postoperative sedation with propofol was associated with delayed time to discharge from PACU.

The intraoperative EtCO_2 _measurements were within normal limits and were comparable in both the groups.

**Figure 7.**
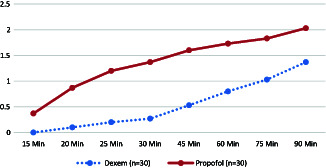
Comparison of mean postoperative VAS score. VAS, visual analogue scale.

Postoperative VAS score was significantly lower with dexmedetomidine as compared to propofol (Figure 7). The overall patient satisfaction was significantly better with dexmedetomidine (*P* ≤ .02).

None of the patients in the study had any clinically significant complication so as to necessitate discontinuation of the infusion of the study drug.

The patient satisfaction score was better with dexmedetomidine infusion as compared to propofol infusion.

## Discussion

In East Asia, up to 30% of all spinal surgery is now performed by endoscopic techniques. The advantages of endoscopy over open procedures include and are not limited to minimal access trauma, less scar formation, reduced blood loss, selective removal of hernia, and protection of spinal canal.^5^

Dexmedetomidine is an attractive agent for short-term procedural sedation and has been safely used in transesophageal echocardiography, colonoscopy, awake carotid endarterectomy, shockwave lithotripsy, vitreoretinal surgery, elective awake fibreoptic intubation, paediatric patients undergoing tonsillectomy, and paediatric magnetic resonance imaging.^6-8^

Propofol, being an ultra-short-acting hypnotic, has obvious advantages over benzodiazepines and opiates, when used for conscious sedation. It ensures a quicker onset of action and less patient discomfort, both of which benefit the endoscopist and the patient. The time to recovery is shorter and hence earlier discharge from the endoscopy unit. Patients who receive propofol (half-life 2-4 minutes) as a single agent recover normal neurological and social functioning significantly quicker than benzodiazepines (half-life 30 minutes) and/or narcotics (half-life 3-4 hours).^9,10^

There is a growing body of evidence supporting the use of propofol in conjunction with small doses of benzodiazepines and/or opiates. With combination therapy, a smaller dose of propofol is required to obtain moderate rather than deep sedation. In a recent study, patient recovery and discharge was faster with combination therapy than with propofol alone.^10,11^ We therefore used midazolam and fentanyl as premedication in the present study.

From 30 minutes of start of the infusion, the heart rate was significantly lower with dexmedetomidine as compared to propofol. This difference persisted in the postoperative period and can be explained to the sympatholytic and vagomimetic properties of dexmedetomidine. Similar results have been demonstrated by other authors.^12,13^

Propofol, due to its direct powerful inhibitory effect on sympathetic outflow, causes vasodilatation leading to a fall in mean arterial pressure, whereas larger doses of dexmedetomidine have a direct effect at the postsynaptic vascular smooth muscle. We, in our present study, observed significantly higher mean arterial pressure with dexmedetomidine 10 minutes onwards from the start of the infusions. The postoperative values were comparable. These results correlate well with those of Shah et al,^12^ Mahmoud et al,^13^ Arain and Ebert.^14^

All the patients in our study achieved the desired levels of sedation. The sedation levels however were more rapidly achieved with propofol (10 minutes), as compared to dexmedetomidine (20 minutes). This is attributed to high lipophilicity of propofol and hence its rapid distribution in the central nervous system. Similar results were obtained by various studies.^12,14,15^ Propofol provided a deeper level of sedation than dexmedetomidine as observed by both Ramsay sedation score as well as BIS values. This is consistent with most of the earlier trials performed.^12,14,16^

Both propofol and dexmedetomidine are known to have minimal respiratory depression when used as sedative agents which is evident for our results, wherein there was no desaturation or a fall in the respiratory rate from baseline in either of the groups.^12^

Intraoperative dexmedetomidine infusion provided significantly lower postoperative VAS scores as compared to propofol, which also reflected as better patient satisfaction. Dexmedetomidine has proven analgesic properties, in contrast to propofol. Its half life of 2 hours extends the analgesic sparing benefits in the postoperative period as well.^12,17,18^

The recovery time, as measured by the Aldrete score, was significantly more with dexmedetomidine than with propofol due to deeper level of postoperative sedation seen with the former. The patients however were easily arousable. These observations are comparable with previous studies.^12,14,18^

One of the limitations of our study was small sample size, but it had significantly important results, and we suggest future studies to be undertaken with a larger population size. Also, we did not document the incidence of recall in our study. We however have compared patient satisfaction.

Thus, dexmedetomidine provides better sedation, stable cardiorespiratory profile and analgesic effect, and better overall patient satisfaction.

## Conclusion

Dexmedetomidine and propofol provide adequate levels of sedation without any cardiorespiratory compromise, when used for conscious sedation, for minimally invasive spine surgeries performed in prone position. Dexmedetomidine provides an added advantage of postoperative analgesia.

## Figures and Tables

**Table 1.** Demographic Variables, Baseline Haemodynamic Parameters, and Surgical Duration

Parameter	Group D (n = 30)	Group P (n = 30)	*P*
Age (years)	53.67 ± 5.26	50.23 ± 6.10	.023
Sex (M : F)	19 : 11	18 : 12	.896
Weight (kg)	63.23 ± 5.93	63.2 ± 7.8	.985
HR (min^−1^)	77.40 ± 9.15	77.67 ± 7.19	.901
MAP (mm Hg)	92.57 ± 3.78	91.47 ± 3.98	.277
Duration of surgery (min)	91.67 ± 10.28	91.33 ± 9.99	.899

Values are expressed as mean ± standard deviation.
